# Chemical Composition and Cytotoxicity Evaluation of *Artemisia judaica* L. Essential Oil from Saudi Arabia

**DOI:** 10.3390/molecules29122882

**Published:** 2024-06-18

**Authors:** Bashaer Alsharif, Yasmin Bashir, Fabio Boylan

**Affiliations:** 1 School of Pharmacy and Pharmaceutical Sciences, Panoz Institute, Trinity Biomedical Sciences Institute, Trinity College Dublin, D02 PN40 Dublin, Ireland; alsharib@tcd.ie; 2 Department of Pharmaceutical Sciences, Faculty of Pharmacy, Umm Al-Qura University, Makkah 21955, Saudi Arabia; 3 School of Pharmacy, University of Pavia, 27100 Pavia, Italy; bashirya@tcd.ie; 4 Trinity Centre for Natural Products Research—NatPro, Trinity College Dublin, D02 PN40 Dublin, Ireland

**Keywords:** *Artemisia judaica*, volatile oil, GC–MS, principal component analysis, agglomerative hierarchical cluster

## Abstract

Gas chromatography (GC) and gas chromatography–mass spectrometry (GC–MS) analyses were conducted on essential oil extracted from Saudi Arabian *Artemisia judaica* L. (*A. judaica*) aerial parts, resulting in the identification of 58 constituents, representing 93.0% of the total oil composition. The oil primarily consisted of monoterpenes (38.6%), sesquiterpenes (14.1%), and other compounds such as ethyl esters and cyclic ketones (40.3%). The main components identified were piperitone (16.5%), ethyl cinnamate (12.9%), and camphor (9.7%). Multivariate statistical analyses (MVAs), including principal component analysis (PCA) and agglomerative hierarchical clustering (AHC) analysis, were employed to compare the chemical makeup of this oil with 20 other *A. judaica* oils from various regions. The study revealed distinct clusters, highlighting unique chemotypes and geographic variations. Particularly, the oil from the current study demonstrated a specialized chemical profile with significant concentrations of specific compounds, contributing significantly to its distinctiveness. Further cytotoxicity testing on RAW264.7 macrophages suggested that concentrations below 20 μg/mL of *A. judaica* oil are suitable for future pharmacological investigations. This study provides valuable insights into the chemical diversity, geographic variations, and potential biomedical applications of these essential oils.

## 1. Introduction

*Artemisia* is one of the largest and most diverse genus within the Asteraceae family. It comprises approximately 500 species distributed worldwide and is known for its aromatic characteristics. The species within this genus are well recognized for their bitter taste, mainly attributed to the terpenoids and sesquiterpene lactones they contain [1]. The genus encompasses a variety of herbs and small shrubs that have been utilized in folk and traditional medicine for various purposes, such as respiratory disorders, allergies, hypertension, and others [2]. The genus *Artemisia* also holds significance in the industry for its diverse range of properties, including its effectiveness against microbes and insects, its antioxidant capabilities, and its role in combating malaria, along with its contribution to perfumery through the production of aromatic compounds [3].

*A. judaica* L. is one of the species within the *Artemisia* genus, specifically found in Mediterranean countries such as Saudi Arabia, Egypt, Algeria, Libya, and Jordan [4]. Thriving in desert environments, particularly sandy wadi beds, this perennial, aromatic shrub reaches heights of 20–30 inches. Characterized by a bushy growth habit and a woody base, *A. judaica* bears yellowish flowers atop a short stem. Fine hairs cover its branches, and its leaves, arranged alternately, are short, grayish, and hairy and feature dissected rounded heads [5,6].

Traditionally, this plant is known by different local names, such as Shih in Saudi Arabia, Beithran in Jordan, or Teheregle in Algeria, and has been used in folk medicine for the treatment of various diseases. Some traditional uses of *A. judaica* include treating gastrointestinal problems, skin diseases, and atherosclerosis, acting as an immune stimulant, reducing fever and stress, and regulating menstruation [7]. It has also been traditionally consumed as a tea in Egypt or used as a food condiment (due to its strong fragrance) in many places in the Middle East and Africa [8].

Chemical analysis of *A. judaica* revealed the presence of flavonoids, phenolics, triterpenes, and sesquiterpene lactones such as judaicin [9]. Additionally, *A. judaica*, being an aromatic plant, has its volatile constituents identified from various geographical regions and climates. Apart from anthropogenic factors, environmental conditions play a significant role in determining the volatile composition of the plant [10]. The overall analysis of *A. judaica* essential oil constituents indicates that the monoterpene piperitone is the primary constituent across different genotypes [11,12]. Moreover, other chemical constituents, such as camphor, ethyl cinnamate, and spathulenol, have been found in relatively high concentrations in specific plant genotypes [11]. Furthermore, environmental factors and geographical locations have been observed to influence the major constituents of the essential oil from different *A. judaica* [4].

Therefore, this study aimed to conduct a detailed analysis (using GC and GC–MS) of the essential oil extracted from *A. judaica* collected from Saudi Arabia. The study also aimed to compare its chemical composition with oils from other *A. judaica* samples across various geographic locations, employing multivariate statistical analyses such as agglomerative hierarchical clustering (AHC) analysis and principal component analysis (PCA). Considering that monoterpene diversity and morphology can reflect gene flow patterns [13], the primary objective of the multivariate analyses was to gain insights into the heredity of chemical traits within this species. Furthermore, the secondary objective of this study was to assess the cytotoxic effects of *A. judaica* essential oil on Raw 264.7 macrophage cells.

## 2. Results

**Figure 1:**
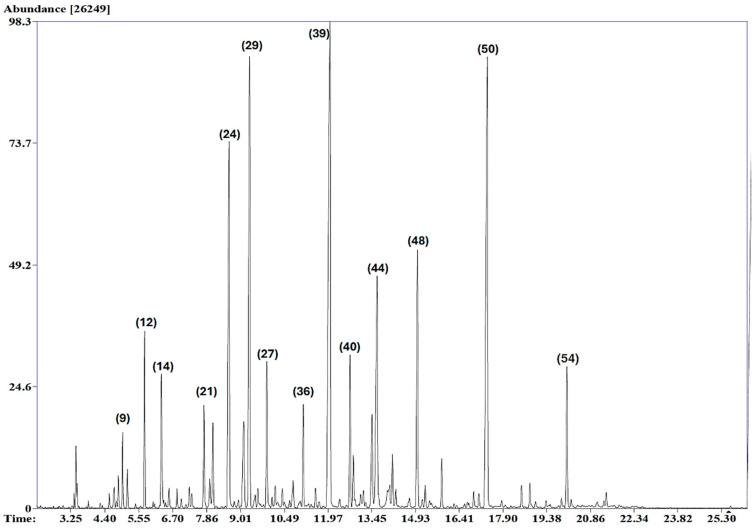
Total Ion Chromatogram (TIC) of the essential oil of *A. judaica*.

**Figure 2:**
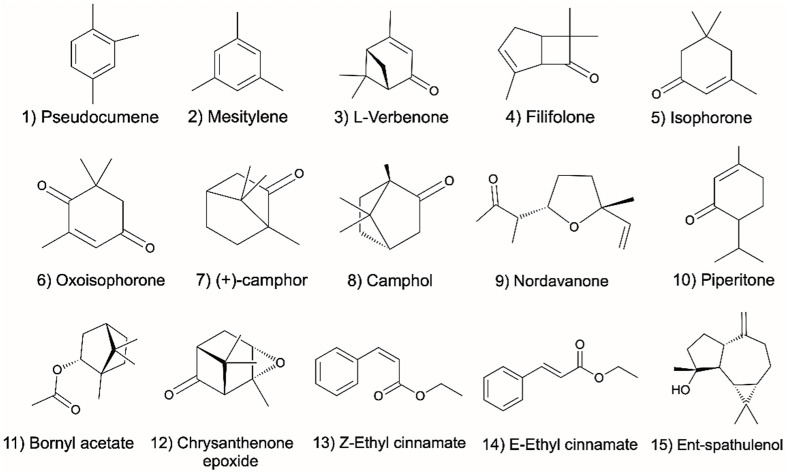
Chemical structures of the main compounds identified in *A. judaica* essential oil.

Hydrodistillation of the aerial parts of *A. judaica* yielded a yellow oil with a light woody fragrance. GC and GC–MS analyses allowed for the identification of 58 compounds, comprising 92.7% of the total peak areas. The oil was dominated by monoterpenes (M; 38.6%), followed by sesquiterpenes (S; 14.1%) and other compounds (O; 40.33%), such as ethyl esters and cyclic ketone. The major components of the oil were identified as piperitone (**39**; 16.5%), ethyl cinnamate (**50**; 12.9%), and camphor (**27**; 9.7%). The Total Ion Chromatogram (TIC) of the essential oil of A. judaica is presented in Figure 1. These compounds have already been reported as volatile metabolites of some other Artemisia species [4,6,14]. The other constituents identified in the oil are compiled in Table 1. The chemical structures of the characteristic known compounds found in the analyzed oil are given in Figure 2.

## 3. Discussion

**Figure 3:**
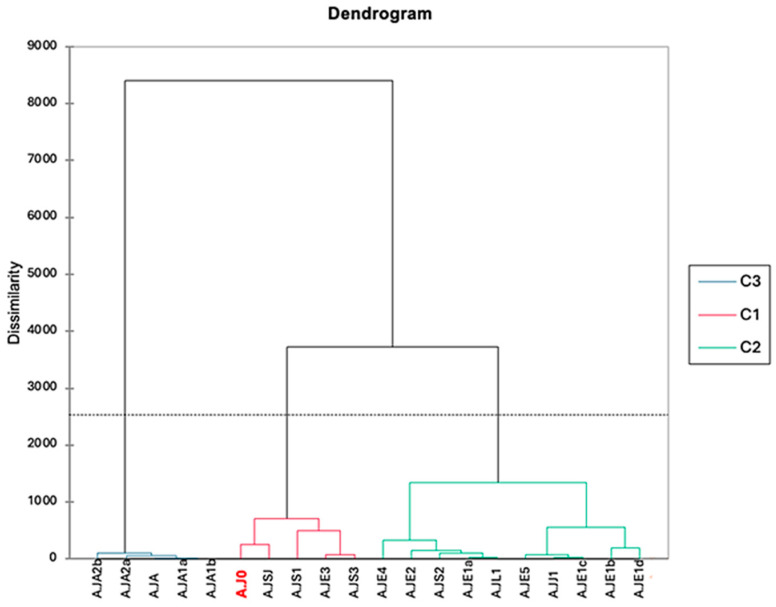
Dendrogram generated from the agglomerative hierarchical clustering (AHC) analysis illustrating the dissimilarity relationships in chemical composition among 20 essential oil samples. The dissimilarities were computed by using Euclidean distance, and Ward’s method was employed as the aggregation criterion.

The AHC dendrogram (Figure 3) reveals three distinct clusters (C1–C3) among the samples (Table 2). Cluster C3 exclusively groups four essential oils (A.JA1a, A.JA2a, A.JA1b, and A.JA2b), all originating from Algeria. This cluster’s distinctiveness stems from the unique chemical profile of its central object, A. JA1a. In AHC, the central object represents the “average” chemical profile within a cluster. A. JA1a exhibits a significantly higher concentration of piperitone (71.1%) compared with Clusters 1 and 2 (samples from Jordan, Saudi Arabia, Egypt, and Libya). Additionally, some compounds, like (+)-camphor, are absent or present in much lower concentrations in Cluster 3 compared with the other clusters. While Clusters 1 and 2 show moderate to high concentrations of (+)-camphor (e.g., Cluster C2: 16.1%), Cluster 3 either lacks or has negligible amounts. This clustering pattern suggests a distinct chemotype or genetic variant of *A. judaica* specific to Algeria, showcasing a consistent and characteristic chemical profile within this subset of samples. The Köppen–Geiger climate classification system can offer insights into why piperitone levels were higher in Algerian samples [15].

Cluster C1 primarily comprises essential oil samples from Saudi Arabia (A.J0, A.JSJ, A.JS1, and A.JS3) and one sample from Egypt (A.JE3), specifically collected from the Matrouh Governorate in North Egypt. Cluster 1 stands out for its relatively lower concentrations of certain compounds compared with the other clusters. For instance, the level of piperitone in Cluster 1 is around 0.5%, indicating a moderate presence of this compound in the essential oils within this cluster. It is worth noting that there is some variation within the cluster, as evidenced by sample AJS0, which has a higher piperitone concentration (16.5%). While piperitone serves as a main constituent contributing to the aroma and potential therapeutic properties of *A. judaica* essential oils [29], its lower concentration in Cluster 1 suggests a unique chemical profile distinct from Clusters 2 and 3.

The unique chemical profile observed in Cluster 1 (C1) of *A. judaica* samples, obtained from Saudi Arabia and Egypt’s Matrouh Governorate, is likely influenced by a combination of environmental and genetic factors. The Köppen–Geiger climate classification system identifies both regions as predominantly hot desert climates (BWh/BSh) [30], which may induce the production of specific volatile compounds like camphor, known for their potential roles in heat stress tolerance [31,32], herbivore deterrence [33], and pollinator attraction [34]. Furthermore, variations in soil composition and altitude within the C1 regions, as well as genetic variability within and between *Artemisia* populations, could contribute to the observed chemical diversity [35,36,37].

All remaining samples from Egypt, Saudi Arabia, Libya, and Jordan, excluding those already classified in Cluster C3 and Cluster C1, were deemed statistically not different and subsequently grouped within Cluster C2. Cluster 2 also demonstrates unique characteristics based on the central object A.JJ1 in the provided table. A.JJ1’s chemical composition profile differs notably from the central objects of Clusters 1 and 3. Specifically, A.JJ1 exhibits a distinct concentration of chemical compounds such as (+)-camphor, piperitone, chrysanthenone epoxide, z-ethyl cinnamate, and e-ethyl cinnamate, which are either absent or present in lower concentrations in the central objects of Clusters 1 and 3. Conversely, compounds like filifolone and ent-spathulenol, prominent in Clusters 1 and 3, are notably low in A.JJ2, highlighting a specific chemical composition profile that sets it apart from the other clusters analyzed. This clustering outcome suggests that these samples share similar volatile composition, indicating potential chemical similarity or comparable chemotypes across these regions. The Köppen–Geiger climate classification system can provide context for this grouping [15], as regions with similar climatic conditions, such as hot desert climates (BWh/BSh) [30], prevalent in these countries, may foster similar chemical profiles in *A. judaica* populations. Factors such as environmental stressors, soil characteristics, and genetic variability within the plant species may also contribute to the observed chemical similarities among samples in Class C2, despite their geographic diversity [37,38,39].

**Figure 4:**
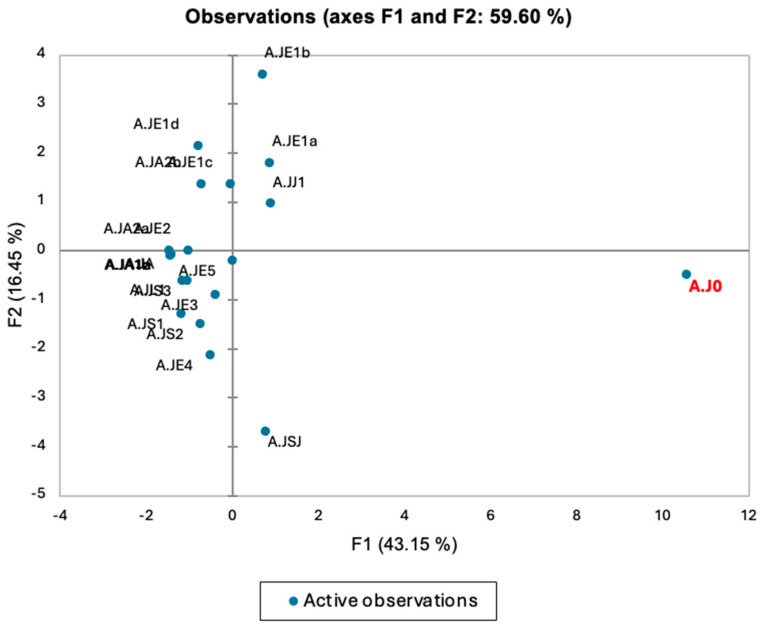
Ordination plot resulting from principal component analysis (PCA) applied to 20 oil samples. Axes F1 and F2 correspond to the first and second principal components, respectively. Axis F1 explains approximately 43.2% of the total variance, while axis F2 contributes an additional 16.5%.

The PCA conducted on the *A. judaica* essential oil dataset revealed insightful patterns and relationships among the chemical compounds present in the samples (Figure 4). One particularly unique observation, AJ0, stood out in the analysis. Positioned in the lower right quadrant of the PCA plot, AJ0 exhibited distinct characteristics compared with the other samples. This distinctiveness can be attributed to its high values or concentrations of key chemical compounds contributing significantly to the F1 dimension, namely, pseudocumene, l-verbenone, oxoisophorone, and nordavanone. However, piperitone levels appeared to play a particularly crucial role. High piperitone concentration in AJ0, especially compared with other Saudi samples, likely explains its outlier status in the PCA plot. The analysis suggests that variations in piperitone levels are key to understanding AJ0’s unique chemical makeup within the context of *A. judaica* essential oils.

While the PCA revealed a unique sample (AJ0) with high piperitone concentration, *A. judaica*, in general, seems to be dominated by this monoterpene, found across various plant families. The dominance of piperitone in *A. judaica* suggests a potentially significant role in the overall biological activity of the oil. While first discovered in eucalyptus oil in 1907 [40], piperitone is also prevalent within the mint family (*Labiatae*), particularly the genus *Mentha* [41]. Interestingly, piperitone exists in two isoforms, and the specific ratio of these isomers varies depending on the plant [42]. For *A. judaica*, future research should explore whether the specific makeup of these piperitone isomers contributes to its potential therapeutic effects.

Based on the known properties of piperitone, we can speculate on its potential contributions within *A. judaica*. Piperitone is recognized for its antimicrobial, antifungal, and anti-inflammatory properties [43]. Interestingly, *A. judaica* essential oil itself exhibits similar antifungal and anti-inflammatory activities [6]. This alignment suggests that piperitone might be one of the key contributors to these effects, potentially explaining the traditional use of *A. judaica* oil for treating ailments like gastrointestinal issues and skin conditions, often linked to microbial activity. Furthermore, piperitone exhibits insect repellent qualities [44], which might explain the traditional use of *A. judaica* as a food condiment to repel insects.

Evidence from other *Artemisia* species strengthens the case for the pharmacological potential of piperitone. Abdelgaleil and collaborators have isolated piperitone from *A. herba alba* and demonstrated strong antifungal activity against specific mold strains [29]. This suggests a broader role for piperitone within the *Artemisia* genus, and further investigation is crucial to confirm these possibilities for *A. judaica* essential oil.

**Figure 5:**
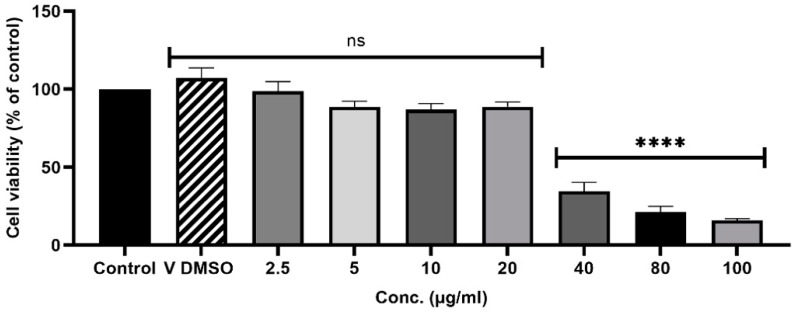
Effect of essential oil of *A. judaica* on macrophages (Raw 264.7) viability by the Alamar blue assay. Negative Ctrl (control); tested concentrations of the essential oil (2.5–100 µL/mL). Results are expressed as percentages of Alamar blue reduction by control cells. Each value represents the mean ± SD of three independent experiments performed in duplicate (**** *p* < 0.0001, compared with control). ns = not statistically significant.

*A. judaica* oil (AJ0) exhibited concentration-dependent cytotoxicity on RAW264.7 macrophages (Figure 5). Concentrations up to 20 µg/mL showed no significant decrease in cell viability, while higher concentrations were cytotoxic. There is a limited number of studies on *A. judaica* oil cytotoxicity and in vitro anti-inflammatory activity. Most papers are either conducted on the *A. judaica* extract or test the oil in vivo. However, there is a previous study reporting no cytotoxicity of *A. judaica* oil towards macrophages (Raw 264.7) and hepatocytes (HepG2) at concentrations up to 1.0 µg/mL. Interestingly, this study also observed decreased NO production in macrophages with the increase in oil concentration, suggesting potential anti-inflammatory properties [6].

To understand the cytotoxic mechanism at high concentrations, future studies could investigate pathways like apoptosis or oxidative stress. For therapeutic applications, especially those targeting anti-inflammatory or immunomodulatory effects, concentrations below 20 µg/mL are recommended. Further research should identify specific bioactive compounds responsible for cytotoxicity and explore the oil’s therapeutic potential by using primary cells or animal models.

## 4. Materials and Methods

### 4.1. Chemicals and Reagents

Dimethyl sulfoxide (DMSO), of cell culture grade (99.9% purity), was purchased from Panreac (Barcelona, Spain). Dulbecco’s Modified Eagle’s Medium (DMEM) high in glucose was purchased from Sigma Aldrich (Saint Louis, MO, USA)*,* and fetal bovine serum (FBS) was purchased from Gibco Biosciences (Miami, FL, USA). The Alamar Blue assay kit was purchased from Thermo Fisher Scientific, Waltham, MA, USA.

### 4.2. Plant Material

Samples *of A. judaica* were collected from Jabal Al-Lawz, Saudi Arabia, in November 2022. The plant was identified by Prof. Ammar Bader, and a voucher specimen (SA-AJ 2019-2) was deposited in the herbarium of the pharmacognosy lab, Umm Al-Qura University.

### 4.3. Essential Oil Extraction

Aerial parts of *A. judaica* (100 g) were mixed with 1000 mL of distilled water and subjected to steam distillation for 3 h at 100 °C by using a Clevenger apparatus (Cruinn Diagnostics Ltd., Dublin, Ireland) as described by Ornano et al. [45]. During the process, the essential oils, vaporized and carried by the steam, were condensed and separated from the water. The yield of the essential oils was 0.7% (*v*/*w*), calculated as the volume (mL) obtained per gram of plant material. The resulting pale-yellow essential oil with a characteristic odor was stored at 4 °C in an amber vial until further analysis.

### 4.4. Chromatographic Analysis

The GC–MS analysis was conducted in triplicate by using a Hewlett-Packard 6890N gas chromatograph equipped with a DB-5MS fused-silica capillary column (5% phenylmethylsiloxane, 30 m × 0.25 mm, film thickness of 0.25 μm; Agilent Technologies Inc., Santa Clara, CA, USA) and coupled with a 5975B mass selective detector from the same company as described by [46]. The apparatus was set up to operate at 70 eV over a mass range of 35–500 amu and a scanning speed of 0.34, and it was equipped with a DB-5MS fused-silica capillary column (Agilent Technologies, USA). The temperature was regulated to increase by 5 °C per minute, allowing a gradual increase from 70 °C to 290 °C. The injector temperature was 250 °C, the interface temperature was 300 °C, and the carrier gas was helium (1.0 mL/min). Each ml of oil was dissolved in Et2O (1:1000 ratio) and then injected in pulsed split mode. The split ratio was 40:1, and the flow rate was initially set to 1.5 mL/min for the first thirty seconds and then adjusted to 1.0 mL/min for the remainder of the run. The GC (FID) studies mirrored the GC–MS conditions. No correction factors from the GC peak areas were employed in the calculation of the percentage composition.

### 4.5. Compound Identification

The identification of the essential oil compounds was achieved by (I) experimentally determining their linear retention indices (RI) on a DB-5MS column by using n-alkanes (C8-C40) (Agilent Technologies, USA) as reference points and comparing them with published RI values [47] and (II) tentatively identifying compounds by cross-referencing mass spectra data with established MS libraries, including Wiley 6, NIST02, and NIST 17 [48].

### 4.6. Multivariate Statistical Analyses (MVAs)

The essential oil components of 22 *Artemisia* samples, including the current study, were collectively analyzed by using multivariate analyses (MVAs), specifically principal component analysis (PCA) and agglomerative hierarchical clustering (AHC). AHC and PCA were conducted by using the Excel program plugin XLSTAT, version 2021.1.1. Both statistical methods utilized the percentages of individual oil constituents as variables, considering only those constituents with a percentage higher than 1% in at least one sample. AHC employed Pearson dissimilarity with aggregation criteria including simple linkage, unweighted pair-group average, and complete linkage, along with Euclidean distance utilizing aggregation criteria such as weighted pair-group average, unweighted pair-group average, and Ward’s method. A PCA of the Pearson (n) type was also carried out.

### 4.7. Cytotoxicity Assay

#### 4.7.1. Cell Culture and Treatment

Murine macrophages (RAW264.7 cells) were cultured in DMEM high in glucose, supplemented with 10% fetal bovine serum (FBS), at 37 °C in a 5% CO_2_ incubator. Once RAW264.7 cells reached 80% confluency, they were detached and seeded in 96-well plates (1 × 10^5^ cells/well). After 24 h, cells were treated with *A. judaica* oil at concentrations of 100, 80, 40, 20, 10, 5, and 2.5 µg/mL for 24 h.

#### 4.7.2. Cell Viability Assay

The Alamar Blue assay was used to quantitatively assess RAW264.7 cell viability after exposure to the *A. judaica* oil, following the manufacturer’s instructions. Briefly, following incubation with various concentrations of the oil or the DMSO vehicle diluted in serum-free culture medium for 24 h, the medium was removed, and cells were incubated for another 2 h in the presence of 2% Alamar Blue dye. Post-incubation, fluorescence was recorded at wavelengths of 560 nm (excitation) and 590 nm (emission) by using Fluorstar Optima, BMG labtech, Germany.

### 4.8. Statistical Analysis

All statistical analyses were conducted by using GraphPad Prism 9.0 software (GraphPad Software, San Diego, CA, USA). The results are presented as means ± standard deviation (SD) from at least three independent experiments, each performed in triplicate. Statistical significance between groups was determined by using one-way ANOVA followed by Dunnett’s or Duncan’s multiple comparison post hoc test, as appropriate. A *p*-value < 0.05 or <0.01 was considered statistically significant.

## 5. Conclusions

In conclusion, multivariate analyses of *A. judaica* essential oil revealed distinct chemical profiles based on geographic origin, with sample AJ0 being identified as an outlier due to its unique composition. These findings highlight the importance of considering both geographic origin and chemical composition when studying essential oils and their potential applications. Cytotoxicity assays on murine macrophages indicated that *A. judaica* essential oil is safe for further pharmacological research at concentrations below 20 µg/mL. Future investigations should further explore the biological potential of *A. judaica* essential oil.

## Figures and Tables

**Table 1:** Composition of the aerial parts’ essential oil of *A. judaica* from Saudi Arabia.

Compounds	Peak Area %	Retention Time	Retention Index ^(a)^	Class ^(b)^	Identification
5-Tert-Butyl-1,3 Cyclopentadiene (**1**)	0.2	3.4	837	O	GC–MS
Ethyl-2-Methylbutanoate (**2**)	0.6	3.4	843	O	GC–MS
Ethyl Isovalerate (**3**)	0.3	3.5	846	O	GC–MS
2,6-Dimethyl-1,5-Heptadiene (**4**)	0.1	3.8	881	M	GC–MS
5-Ethyl-5-Methyl-5-Vinyl Tetrahydrofuran (**5**)	0.1	4.2	912	O	GC–MS
Tricyclene (**6**)	0.2	4.6	931	O	GC–MS
Propyl-2-Methylbutanoate (**7**)	0.4	4.7	941	O	GC–MS
Propyl Isovalerate (**8**)	0.1	4.8	945	O	GC–MS
Camphene (**9**)	0.9	5.0	955	O	GC–MS
Benzaldehyde (10)	0.5	5.2	952	O	GC–MS
6-Methyl-5-Hepten-2-One (**11**)	0.1	5.4	984	M	GC–MS
Pseudocumene (**12**)	2.4	5.8	1002	O	GC–MS
*O*-Allyltoluene (**13**)	0.1	6.0	1015	O	GC–MS
Mesitylene (**14**)	2.4	6.3	1028	O	GC–MS
6-Methyl-5-Octen-2-One (**15**)	0.1	6.4	1032	M	GC–MS
Lavender Lactone (**16**)	0.3	6.6	1039	O	GC–MS
*cis*-Arbusculone (**17**)	0.3	6.9	1052	O	GC–MS
2,6-Dimethyl-1,5-Heptadien-3-Ol (**18**)	0.2	7.0	1059	O	GC–MS
*trans*-Arbusculone (**19**)	0.3	7.3	1071	O	GC–MS
1-Isopropyl-3-Methylenecyclohexane (**20**)	0.2	7.3	1075	O	GC–MS
*L*-Verbenone (**21**)	1.7	7.8	1094	M	GC–MS
Dehydrosabinaketone (**22**)	0.6	8.0	1103	S	GC–MS
Filifolone (**23**)	1.5	8.1	1107	M	GC–MS
Isophorone (**24**)	8.6	8.6	1129	O	GC–MS
1,3,3-Trimethylcyclohex-1-Ene-4-Carboxaldehyde (**25**)	0.2	8.9	1141	O	GC–MS
Oxoisophorone (**26**)	2.3	9.1	1156	O	GC–MS
(+)-Camphor (**27**)	9.7	9.3	1141	M	GC–MS
*cis*-Chrysanthenol (**28**)	0.3	9.6	1168	M	GC–MS
Camphol (**29**)	2.6	9.9	1184	M	GC–MS
4-Terneol (**30**)	0.2	10.1	1188	S	GC–MS
*P*-Cymen-8-Ol (**31**)	0.4	10.2	1192	M	GC–MS
Cryptone (**32**)	0.2	10.3	1195	M	GC–MS
(+)-A-Terpineol (**33**)	0.3	10.4	1248	M	GC–MS
Verbenone (**34**)	0.7	10.8	1263	M	GC–MS
*trans*-Carveol (**35**)	0.2	11.0	1272	M	GC–MS
Nordavanone (**36**)	1.7	11.1	1276	S	GC–MS
Ethyl Phenylacetate (**37**)	0.4	11.5	1293	O	GC–MS
Cuminal (**38**)	0.1	11.7	1298	O	GC–MS
Piperitone (**39**)	16.5	12.0	1249	M	GC–MS
Bornyl Acetate (**40**)	2.6	12.7	1313	M	GC–MS
Thymol (**41**)	1.0	12.8	1345	M	GC–MS
Carvacrol (**42**)	0.3	13.1	1354	M	GC–MS
*cis*-Methyl Cinnamate (**43**)	0.4	13.2	1358	M	GC–MS
Chrysanthenone Epoxide (**44**)	6.4	13.6	1377	S	GC–MS
Piperitenone (**45**)	1.0	14.2	1398	S	GC–MS
Ethyl Dihydrocinnamate (**46**)	0.4	14.3	1402	O	GC–MS
Capric Acid (**47**)	0.3	14.7	1421	O	GC MS
*Z*-Ethyl Cinnamate (**48**)	5.1	15.0	1433	O	GC–MS
Davanafuran (**49**)	0.8	15.8	1467	S	GC–MS
*E*-Ethyl Cinnamate (**50**)	12.9	17.4	1543	O	GC–MS
Artedouglasia Oxide D (**51**)	0.4	18.5	1581	S	GC–MS
Artedouglasia Oxide A (**52**)	0.5	18.8	1593	S	GC–MS
Laciniata Furanone H (**53**)	0.1	19.0	1601	O	GC MS
Artedouglasia Oxide C (**54**)	0.1	19.4	1618	S	GC–MS
Artedouglasia Oxide B (**55**)	0.1	11.7	1641	S	GC–MS
Ent-Spathulenol (**56**)	16.5	12.0	1650	S	GC–MS
Caryophyllene Oxide (**57**)	2.6	12.7	1706	S	GC–MS
Methyl Jasmonate (**58**)	1.0	12.8	1710	O	GC–MS
**Total**	93.0				
**Monoterpenes** (**M**)	38.6				
**Sesquiterpenes** (**S**)	14.1				
**Others** (**O**)	40.3				

^(a)^ RI: retention index experimentally determined on a DB-5MS column relative to the Rt of n-alkanes (C8–C40); the compounds are listed in the order of elution. ^(b)^ Compound identification: RI and mass spectra mass spectra (MS) data compared against commercially available MS libraries Wiley 6, NIST02, and NIST 17.

**Table 2:** List of *A. judaica* essential oil samples used for statistical analysis.

Taxon	Plant Part	Origin	Location	Code	Reference
1. ***A. judaica***	Aerial parts	Saudi Arabia	Jabal el Lawz	A.J0	Present study
2. ***A. judaica***	Aerial parts	Jordan	Al-Mudawarth	A.JJ1	[6]
3. ***A. judaica***	Aerial parts	Saudi Arabia	Northern region	A.JS1	[3]
4. ***A. judaica***	Aerial parts	Saudi Arabia	Northern Qassim region	A.JS2	[4]
5. ***A. judaica***	Aerial parts	Egypt	Sinai	A.JE1a	[16]
6. ***A. judaica***	Aerial parts	Egypt	Gabal Ataka, Suez desert	A.JE2	[17]
7. ***A. judaica***	Aerial parts	Saudi-Jordan Border	Near Saudi-Jordan borders	A.JSJ	[1]
8. ***A. judaica***	Aerial parts	Egypt	Matrouh Governorate	A.JE3	[18]
9. ***A. judaica***	Aerial parts	Saudi Arabia	Riyadh region	A.JS3	[11]
10. ***A. judaica***	Aerial parts	Egypt	Noarth Coast	A.JE4	[19]
11. ***A. judaica***	Aerial parts	Egypt	Saint Catherine in South Sinai	A.JE1b	[20]
12. ***A. judaica***	Aerial parts	Algeria	Tassili n’Ajjer	A.JA1a	[21]
13. ***A. judaica***	Aerial parts	Egypt	El Sharkia Governorate	A.JE5	[22]
14. ***A. judaica***	Aerial parts	Algeria	Weddi tasset in Illizi	A.JA2a	[23]
15. ***A. judaica***	Aerial parts	Algeria	Tamenerast desert, south Algeria	A.JA1b	[24]
16. ***A. judaica***	Aerial parts	Libya	Western Hamada	A.JL1	[10]
17. ***A. judaica***	Aerial parts	Egypt	El-Arish Region of Sinai Peninsula	A.JE1c	[25]
18. ***A. judaica***	Aerial parts	Egypt	Alexandria state and the Sinai Peninsula	A.JE1d	[26]
19. ***A. judaica***	Aerial parts	Algeria	In Amenas desert, near Illizi city.	A.JA2b	[27]
20. ***A. judaica***	Aerial parts	Algeria	Tamanrasset	A.JA	[28]

## Data Availability

The data presented in this study are available upon request from the corresponding author.
